# Premorbid Use of Statin and Outcome of Acute Ischemic Stroke After Intravenous Thrombolysis: A Meta-Analysis

**DOI:** 10.3389/fneur.2020.585592

**Published:** 2020-11-12

**Authors:** Jia Liu, Qinghai Wang, Chaoqun Ye, Gaifen Li, Bowei Zhang, Zhili Ji, Xunming Ji

**Affiliations:** ^1^China-America Institute of Neuroscience, Xuanwu Hospital, Capital Medical University, Beijing, China; ^2^Department of Cardiology, The Second Hospital, Cheeloo College of Medicine, Shandong University, Jinan, China; ^3^Department of Rehabilitation Medicine, Airforce Medical Center, Air Force Medical University, Beijing, China; ^4^China-America Institute of Neuroscience, Luhe Hospital, Capital Medical University, Beijing, China

**Keywords:** acute ischemic stroke, statin, functional outcome, mortality, symptomatic intracranial hemorrhage, meta-analysis

## Abstract

**Background:** The association between the premorbid use of statin and the early outcomes of acute ischemic stroke (AIS) after intravenous thrombolysis (IVT) remains uncertain. We performed a meta-analysis of observational studies to evaluate the influence of the premorbid use of statin on functional outcome and symptomatic intracranial hemorrhage (SIH) in AIS after IVT.

**Methods:** Relevant studies were identified by search of PubMed, Embase, and Cochrane's Library databases. Only studies with multivariate analyses were included. A random-effect model, incorporating inter-study heterogeneity, was used to pool the results.

**Results:** Twenty observational studies with 20,752 AIS patients who were treated with IVT were included. The pooled results showed that the premorbid use of statin was not associated with improved 3-month favorable functional outcome [odds ratio (OR): 1.05, 95% confidence interval (CI): 0.87–1.26, *p* = 0.60, *I*^2^ = 52%), 3-month functional independence (OR: 1.13, 95% CI: 0.96–1.33, *p* = 0.15, *I*^2^ = 52%), or 3-month mortality (OR: 1.12, 95% CI: 0.94–1.34, *p* = 0.20, *I*^2^ = 20%). Moreover, the premorbid use of statin was associated with an increased risk of SIH in AIS after IVT (OR: 1.48, 95% CI: 1.12–1.95, *p* = 0.006, *I*^2^ = 60%). Subgroup analyses according to study design, adjustment of baseline low-density lipoprotein cholesterol, and definitions of SIH showed consistent results (*p*-values for subgroup difference all >0.05).

**Conclusions:** The premorbid use of statin is not associated with improved functional outcomes or mortality but is associated with a higher risk of SIH in AIS patients after IVT.

## Introduction

Despite significant diagnostic and therapeutic advances in recent decades, acute ischemic stroke (AIS) remains one of the leading causes of morbidity and mortality worldwide (1). Intravenous thrombolysis (IVT) is an effective recanalization strategy in AIS (2), and appropriately applied IVT within the time window of AIS is associated with improved functional outcome and survival in these patients. However, IVT has also been associated with an increased risk of symptomatic intracranial hemorrhage (SIH) in AIS patients, particularly in high-risk patients (2). Statins are a category of cholesterol-lowering medications which have become the cornerstone for the primary and secondary prevention of atherosclerotic cardiovascular diseases (3, 4). Moreover, convincing evidence from pre-clinical studies suggests that the premorbid use of statin is associated with reduced neurological injury after acute cerebral ischemia (4). Although concerns have been raised regarding the potentially increased SIH in AIS patients with premorbid use of statin (5), accumulating evidence from observational studies and meta-analyses showed that the premorbid use of statin in AIS patients may be associated with improved functional outcome and short-term survival (3, 6). Accordingly, recent guidelines for the early management of patients with AIS recommended continuation or early initiation of statin therapy in AIS patients (7). However, these recommendations are mainly based on observational studies of AIS patients with heterogeneous treatments. It remains unknown whether the premorbid use of statin is associated with any clinical benefit in AIS patients after IVT (8). In view of the inconsistent results in previous observational studies (9–28), we aimed to evaluate the influence of the premorbid use of statin on functional outcome and SIH in AIS patients after IVT in a meta-analysis.

## Methods

The meta-analysis was designed and performed in accordance with the Meta-analysis of Observational Studies in Epidemiology (29) and Cochrane's Handbook (30) guidelines.

### Literature Search

The electronic databases of PubMed, Embase, and Cochrane's Library were systematically searched using the combination of the following terms: (1) “statin” OR “3-hydroxy-3-methyl-glutarylCoA reductase inhibitor” OR “CS-514” OR “simvastatin” OR “atorvastatin” OR “fluvastatin” OR “lovastatin” OR “rosuvastatin” OR “pravastatin” OR “pitavastatin” OR “cerivastatin,” (2) “stroke” OR “cerebral infarction” OR “cerebrovascular infarction,” and (3) “thrombolysis” OR “plasminogen activator” OR “thrombolytic” OR “fibrinolysis” OR “urokinase” OR “alteplase” OR “rt-PA” OR “rtPA” OR “t-PA” OR “tPA.” The search was limited to human studies published in English. The reference lists of original and review articles were also analyzed manually. The final literature search was performed on April 20, 2020.

### Study Selection

Studies were included if they met the following criteria: (1) published as full-length article, (2) designed as observational study with longitudinal follow-up, including cohort study, nested case–control study, or *post-hoc* analysis of clinical trials, (3) included patients with AIS that were treated with IVT in accordance with standard protocols, (4) premorbid use of statin at the onset of stroke was identified as exposure, (5) compared the incidence of at least one of the following outcomes between patients with and without the premorbid use of statin, including 3-month favorable functional outcome, 3-month functional independence, SIH, or 3-month all-cause mortality, and (6) reported the adjusted odds ratios (ORs, at least adjusted for age and sex) and their corresponding 95% confidence intervals (CIs) for the above-mentioned outcomes. Favorable functional outcome and functional independence were defined as modified Rankin Scale scores of 0–1 and 0–2, respectively. The diagnosis of SIH was in accordance with the criteria applied in the original studies. In the presence of studies providing estimates for >1 definition, we pooled the estimates of the primary SIH definition as indicated from each included study protocol. Reviews, editorials, pre-clinical studies, and studies irrelevant to the aim of the current meta-analysis were excluded.

### Data Extracting and Quality Evaluation

Literature search, data extraction, and study quality assessment were independently performed by two authors according to the pre-defined inclusion criteria. If inconsistency occurred, discussion with the corresponding author was suggested to resolve the issue. The following data were extracted: (1) name of the first author, publication year, country, and study design, (2) characteristics, numbers, mean age, sex, and the baseline National Institute of Health Stroke Scale (NIHSS) of the patients, (3) time window for IVT, (4) number of patients with premorbid use of statin, (5) follow-up durations, and (6) variables included in the multivariate adjusted studies. The quality of each study was evaluated using the Newcastle–Ottawa Scale (NOS) (31). This scale ranges from 1 to 9 stars and judges the quality of each study regarding three aspects: selection of the study groups, comparability of the groups, and ascertainment of the outcome of interest.

### Statistical Analyses

The associations between the premorbid use of statin and early outcomes in AIS patients after IVT were measured by ORs. To stabilize its variance and normalize the distribution, OR and the standard error from each study were logarithmically transformed (30). Cochrane's *Q*-test was performed to evaluate the heterogeneity among the included studies (30, 32), and the *I*^2^ statistic was also calculated. A significant heterogeneity was considered if *I*^2^ > 50%. A random-effect model was used to pool the results since this model has incorporated the potential heterogeneity of the included studies and therefore could retrieve a more generalized result. Pre-defined subgroup analysis according to study design, adjustment of baseline low-density lipoprotein cholesterol (LDL-C), and definitions of SIH were performed. Potential publication bias was assessed by visual inspection of the symmetry of the funnel plots as well as Egger's regression-test (33). RevMan (Version 5.1; Cochrane Collaboration, Oxford, UK) and STATA software were used for the statistics.

## Results

### Literature Search

**Figure 1:**
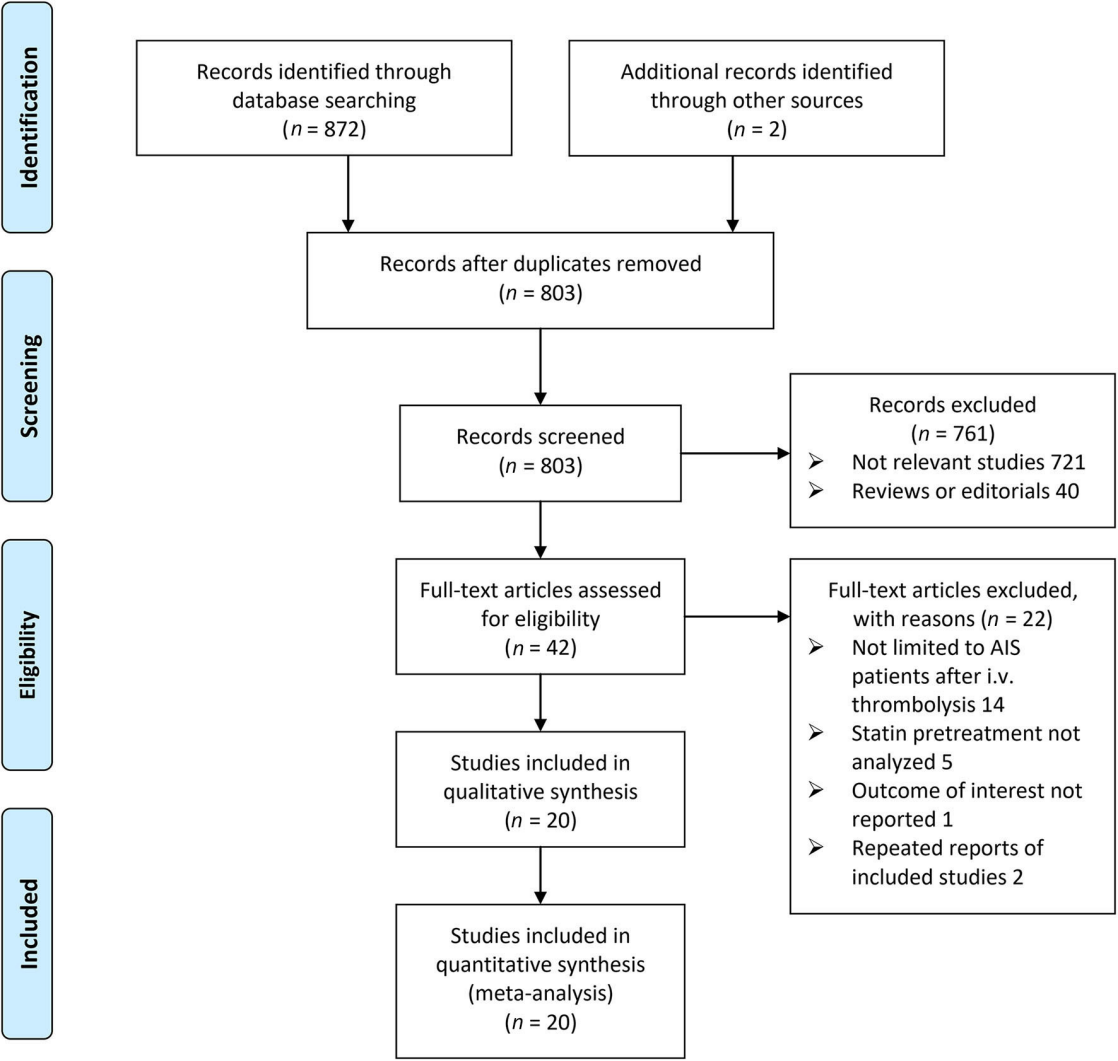
Flow chart of database search and study identification.

The flow chart of the database search is shown in Figure 1. Briefly, 803 studies were obtained from an initial database search after excluding duplications, and 761 of them were subsequently excluded primarily because they were not relevant to the aim of the meta-analysis. For the remaining 42 studies that underwent full-text review, 22 were further excluded for the reasons listed in Figure 1. Finally, 20 follow-up studies were included (9–28).

### Study Characteristics and Quality

The characteristics of the included studies are presented in Table 1. Overall, this meta-analysis included 12 prospective cohort studies (10, 11, 13, 14, 16–18, 20–23, 27), five retrospective cohort studies (9, 12, 15, 19, 26), and three *post-hoc* analyses of RCTs (24, 25, 28), with a total of 20,752 AIS patients who were treated with IVT. The mean ages of the included patients varied between 63 and 75 years, and the baseline NIHSS varied from 7 to 17. Among these patients, 4,097 had premorbid use of statin at the onset of AIS. The AIS patients were followed for 3 months for functional outcomes, SIH, and mortality. Potential confounding factors, including age, sex, baseline NIHSS score, time window for IVT, etiologies of stroke, comorbidities, and concurrent use of antithrombotic medications, were adjusted to a varying degree in the included studies. The quality of the included study was generally good, with the NOS ranging from 7 to 9 (Table 2).

**Table 1:** Characteristics of the included studies.

**Study**	**Country**	**Design**	**Patient number**	**Age**	**Male**	**Baseline NIHSS**	**IV thrombolysis time**	**Patients with premorbid use of statin**	**Follow-up durations**	**Variables adjusted**
				**Years**	**%**				**Months**	
Alvarez 2007	Spain	RC	145	72	52	17	i.v. t-PA in 3 h	26	3	Age, gender, and NIHSS score
Uyttenboogaart 2008	Netherlands	PC	252	68	54	12	i.v. t-PA in 4.5 h	39	3	Age, gender, NIHSS score, LDL-c, early ischemic changes on initial brain CT scan, treatment beyond 3 h, stroke subtype, serum glucose, and use of antiplatelets
Miedema 2009	Netherlands and Belgian	PC	476	69	54	13	i.v. t-PA in 4.5 h	98	3	Age, gender, NIHSS score, hypodensity area on brain CT scan, history of hypertension, DM, DBP, and prior use of antiplatelets
Cappellari 2011	Italy	RC	178	NR	58	NR	i.v. t-PA in 4.5 h	42	3	Age, gender, time window for IV thrombolysis, LDL-c, and stroke severity
Engelter 2011	International	PC	4,012	70	56	12	i.v. t-PA in 4.5 h	918	3	Age, gender, time to thrombolysis, SBP, and stroke severity
Rocco 2012	Germany	PC	1,066	73	53	12	i.v. t-PA in 4.5 h	209	3	Age, gender, baseline NIHSS, hypertension, DM, AF, SBP, glucose level on admission, and stroke etiology
Makihara 2012	Japan	RC	489	71	65	12	i.v. t-PA in 3 h	60	3	Age, gender, blood glucose, LDL-c, baseline NIHSS score, i.v. antihypertensives, occlusion of the internal carotid artery, previous medical histories, and concurrent anti-thrombotic medications
Cougo 2012	Brazil	PC	113	63	52	16	i.v. t-PA in 4.5 h	10	3	Age, gender, and NISS
Martinez 2012	Spain	PC	182	68	54	14	i.v. t-PA in 3 h	30	3	Age, gender, and NISS
Meseguer 2012	France	PC	606	69	57	13	i.v. t-PA in 4.5 h	150	3	Age, sex, treated hypertension, DM, admission glucose level, smoking, and antiplatelet therapy
Cappellari 2013	Italy	RC	2,072	67	58	13	i.v. t-PA in 4.5 h	297	3	Age, sex, time to thrombolysis, baseline NIHSS score, medical histories and concurrent medications
Zhao 2014	China	PC	193	65	36	9	i.v. t-PA in 4.5 h	47	3	Age, gender, and NISS
Scheitz 2014	Germany and Switzerland	PC	1,446	75	54	11	i.v. t-PA in 4.5 h	317	3	Age, gender, LDL-c, NIHSS score, time to thrombolysis, SBP, DM, hypertension, previous stroke, LDL-c, and concurrent anti-thrombotic medications
Scheitz 2015	Germany	PC	481	74	50	11	i.v. t-PA in 4.5 h	83	3	Age, gender, NIHSS on admission, occurrence of pneumonia, history of stroke and AF
Tsivgoulis 2015	International	PC	1,660	67	59	11	i.v. t-PA in 4.5 h	373	3	Age, gender, vascular risk factors, time to thrombolysis, baseline NIHSS scores, admission SBP/DBP
Scheitz 2016	International	*post-hoc* analysis of RCT	2,583	68	57	14	i.v. t-PA in 4.5 h	563	3	Age, sex, baseline NIHSS, AF, previous stroke, onset to treatment time, prior use of antiplatelets, and thrombolysis
				**Years**	**%**				**Months**	
Montaner 2016	Spain	*post-hoc* analysis of RCT	55	74	48	7	i.v. t-PA in 4.5 h	26	3	Age, sex, and baseline NIHSS
Zhao 2017	China	RC	123	66	63	7	i.v. t-PA in 4.5 h	16	3	Age, sex, time to thrombolysis, baseline NIHSS score, onset to treatment time, smoking, and medical histories
Minhas 2018	International	*post-hoc* analysis of RCT	3,284	67	62	8	i.v. t-PA in 4.5 h	615	3	Age, sex, baseline NIHSS, comorbidities, and concurrent anti-thrombotic medications
Erdur 2018	Germany	PC	1,336	75	51	8	i.v. t-PA in 4.5 h	178	3	Age, sex, baseline NIHSS, and glucose level on admission

*IV, intravenous; NIHSS, National Institute of Health Stroke Scale; RC, retrospective cohort; PC, prospective cohort; RCT, randomized controlled trial; t-PA, tissue-type plasminogen activator; CT, computed tomography; DBP, diastolic blood pressure; SBP, systolic blood pressure; DM, diabetes mellitus; AF, atrial fibrillation; LDL-C, low-density lipoprotein cholesterol*.

**Table 2:** Details of study quality evaluation *via* the Newcastle–Ottawa Scale.

**Study**	**Representativeness of the exposed cohort**	**Selection of the non-exposed cohort**	**Ascertainment of exposure**	**Outcome not present at baseline**	**Control for age and gender**	**Control for other confounding factors**	**Assessment of outcome**	**Enough long follow-up duration**	**Adequacy of follow-up of cohorts**	**Total**
Alvarez 2007	0	1	1	1	1	1	1	1	0	7
Uyttenboogaart 2008	1	1	1	1	1	1	1	1	1	9
Miedema 2009	1	1	1	1	1	1	1	1	1	9
Cappellari 2011	0	1	1	1	1	0	1	1	1	7
Engelter 2011	1	1	1	1	1	1	1	1	1	9
Rocco 2012	1	1	1	1	1	1	1	1	1	9
Makihara 2012	0	1	1	1	1	1	1	1	1	8
Cougo 2012	1	1	1	1	1	0	1	1	1	8
Martinez 2012	1	1	1	1	1	0	1	1	1	8
Meseguer 2012	1	1	1	1	1	0	1	1	1	8
Cappellari 2013	0	1	1	1	1	1	1	1	1	8
Zhao 2014	1	1	1	1	1	0	1	1	1	8
Scheitz 2014	1	1	1	1	1	1	1	1	1	9
Scheitz 2015	1	1	1	1	1	0	1	1	1	8
Tsivgoulis 2015	1	1	1	1	1	0	1	1	1	8
Scheitz 2016	0	1	1	1	1	1	1	1	1	8
Montaner 2016	0	1	1	1	1	0	1	1	1	7
Zhao 2017	0	1	1	1	1	0	1	1	1	7
Minhas 2018	0	1	1	1	1	1	1	1	1	8
Erdur 2018	1	1	1	1	1	0	1	1	1	8

### Meta-Analysis Results

**Figure 2:**
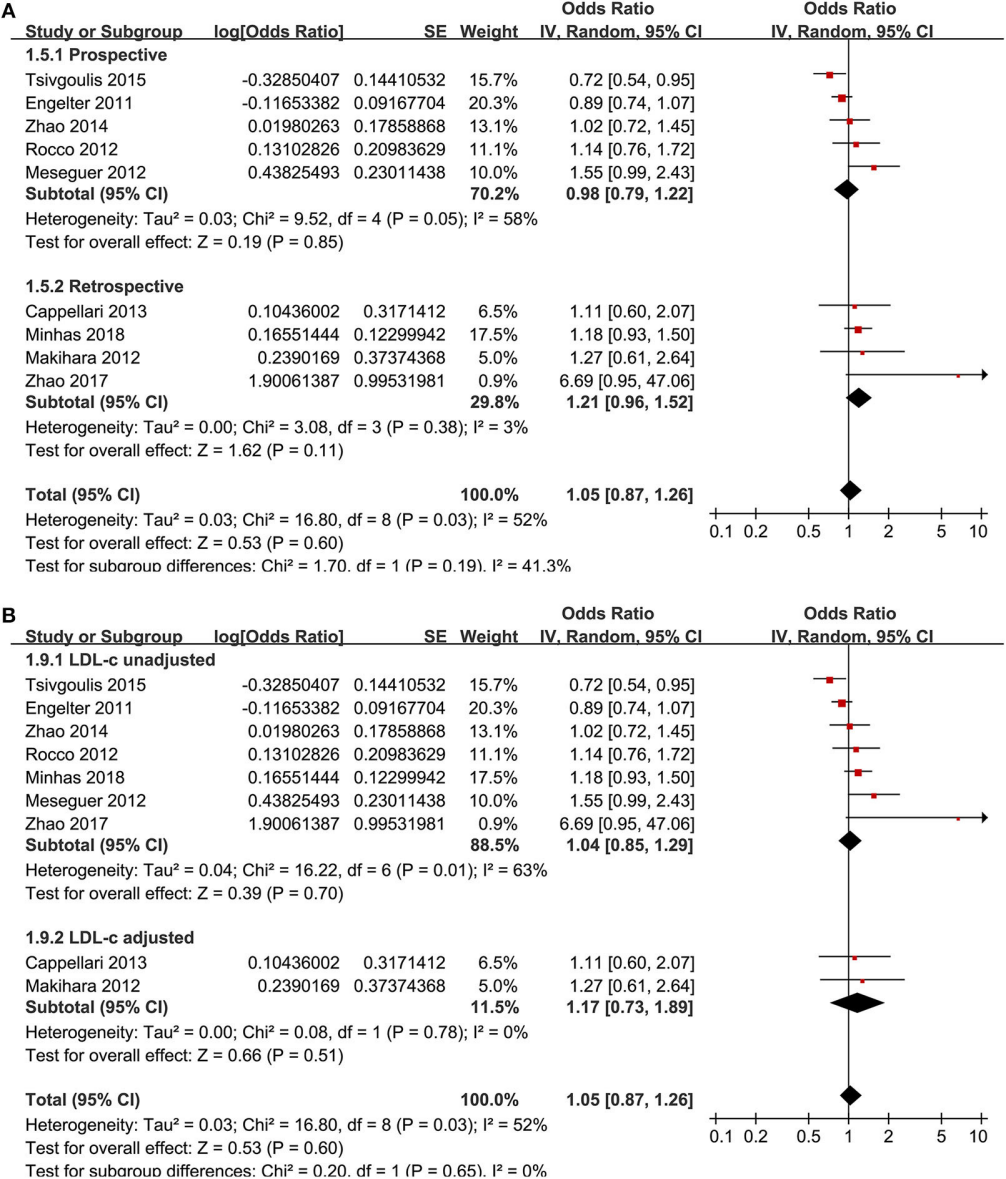
Meta-analysis for the association between the premorbid use of statin and 3-month favorable functional outcome in acute ischemic stroke patients after intravenous thrombolysis. **(A)** Subgroup analysis by study design. **(B)** Subgroup analysis by the adjustment of low-density lipoprotein cholesterol.

Nine studies (13, 15, 17–19, 21, 23, 26, 28) reported the association between the premorbid use of statin and 3-month favorable functional outcome. The heterogeneity was moderate (*p* for Cochrane's *Q*-test = 0.03, *I*^2^ = 52%). The pooled results with a random-effect model showed that the premorbid use of statin was not related to an improved 3-month favorable functional outcome (OR: 1.05, 95% CI: 0.87–1.26, *p* = 0.60; Figure 2) in AIS patients after IVT. Subgroup analyses showed consistent results in prospective or retrospective studies (Figure 2A) and in studies with or without adjustment of LDL-C at baseline (Figure 2B).

**Figure 3:**
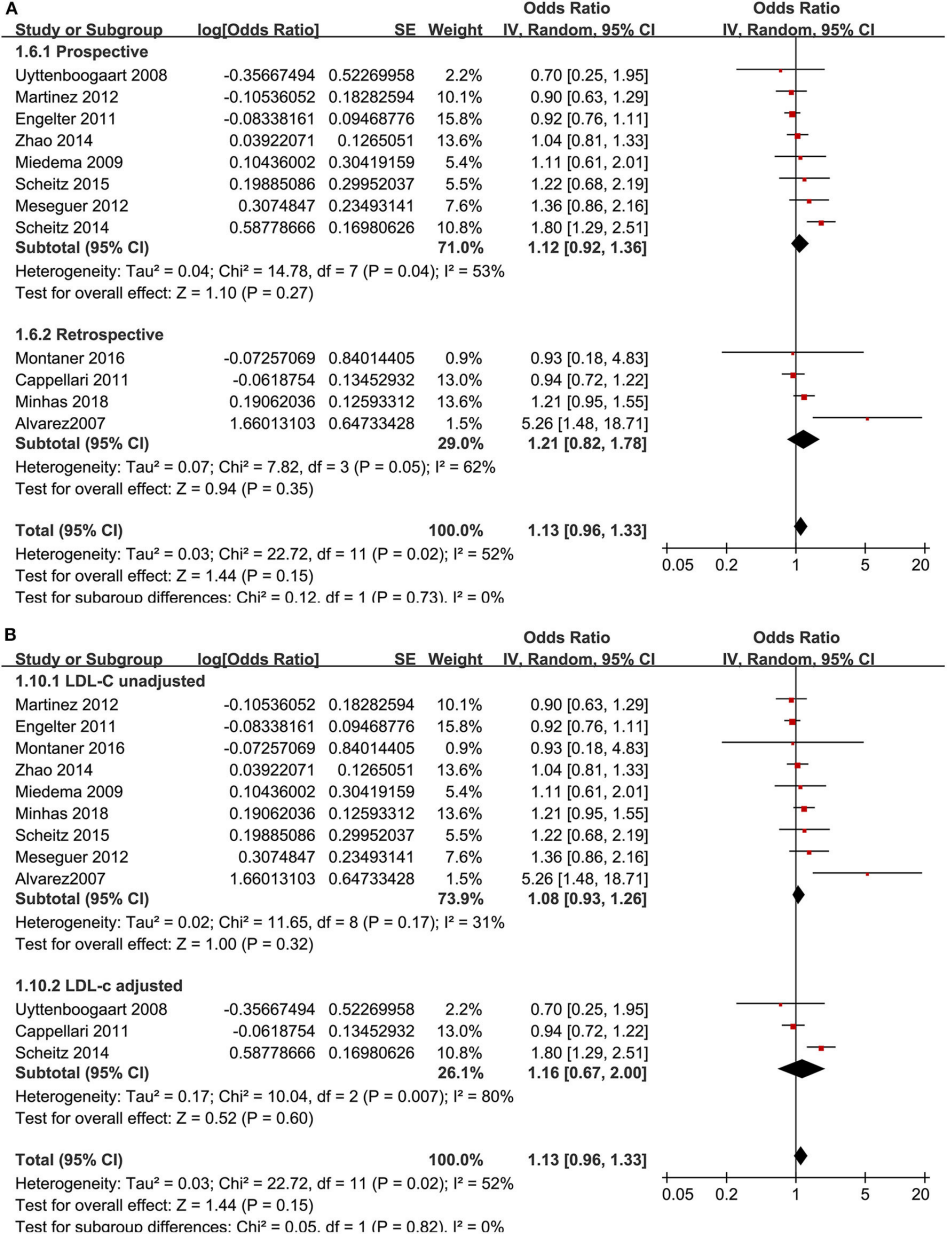
Meta-analysis for the association between the premorbid use of statin and 3-month functional independence in acute ischemic stroke patients after intravenous thrombolysis. **(A)** Subgroup analysis by study design. **(B)** Subgroup analysis by the adjustment of low-density lipoprotein cholesterol.

Similarly, the pooled results with 12 studies (9–13, 16, 17, 20–22, 24, 28) showed that the premorbid use of statin was not related to 3-month functional independence (OR: 1.13, 95% CI: 0.96–1.33, *p* = 0.15, *I*^2^ = 52%; Figure 3), which was consistent in the subgroup analyses according to study design (Figure 3A) or adjustment of LDL-C at baseline (Figure 3B).

**Figure 4:**
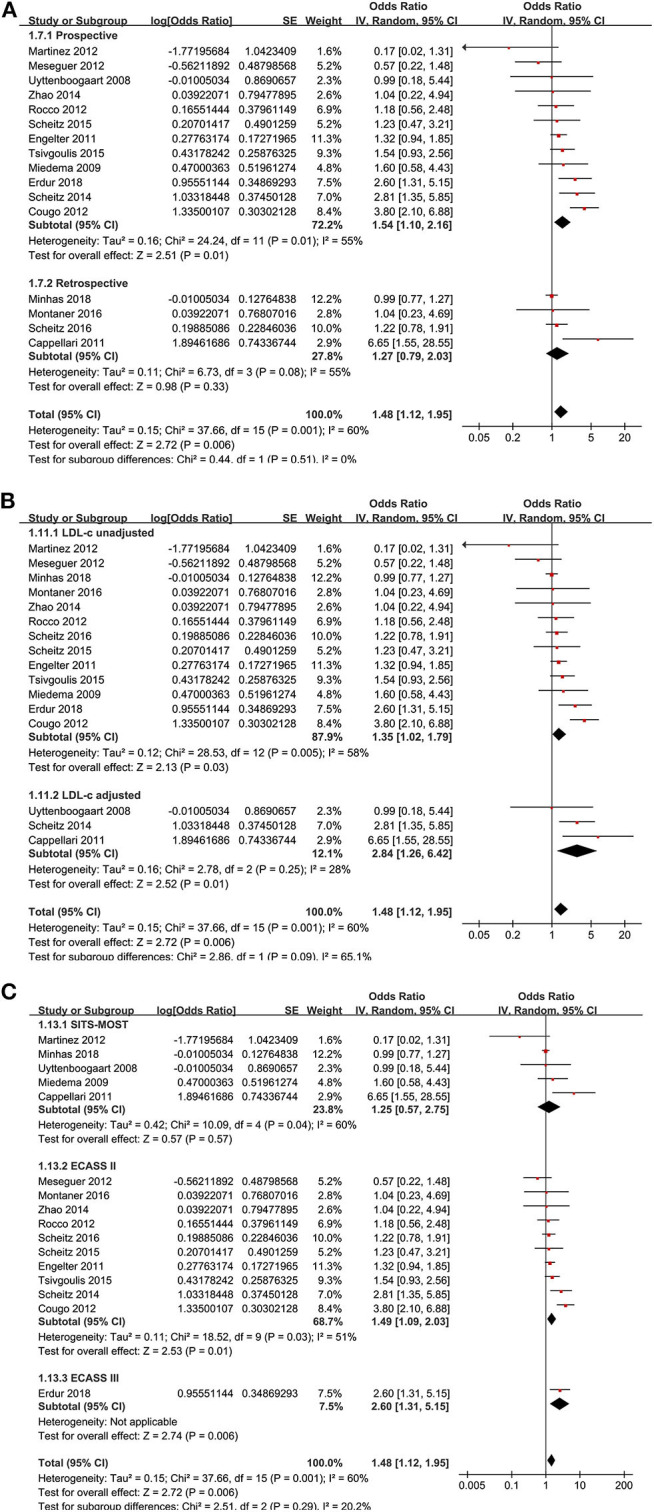
Meta-analysis for the association between the premorbid use of statin and the incidence of symptomatic intracranial hemorrhage (SIH) in acute ischemic stroke patients after intravenous thrombolysis. **(A)** Subgroup analysis by study design, **(B)** subgroup analysis by the adjustment of low-density lipoprotein cholesterol, and **(C)** definitions of SIH.

**Figure 5:**
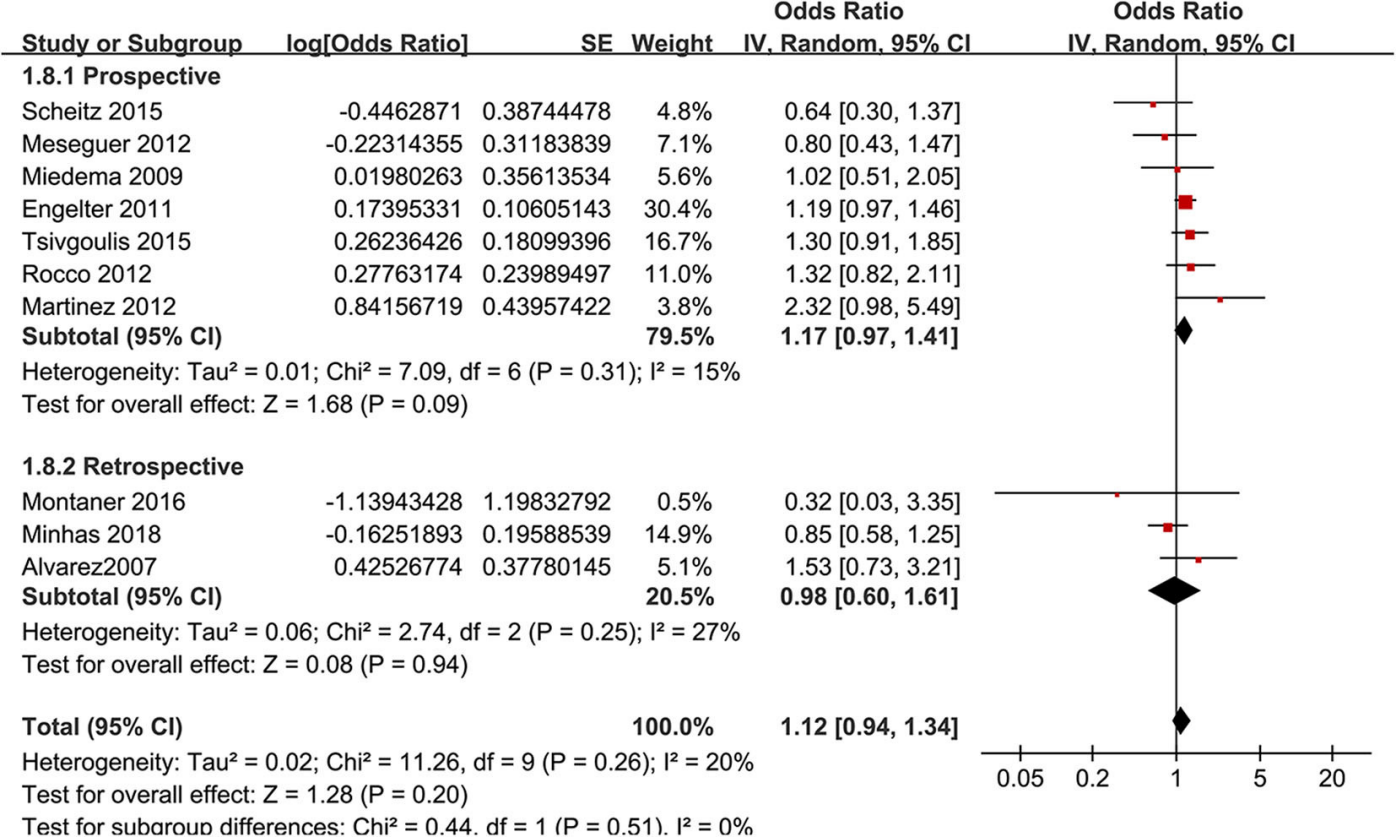
Meta-analysis for the association between the premorbid use of statin and 3-month all-cause mortality in acute ischemic stroke patients after intravenous thrombolysis by study design.

Moreover, the premorbid use of statin was associated with an increased risk of SIH in AIS after IVT [16 studies (10–14, 16–18, 20–25, 27, 28), OR: 1.48, 95% CI: 1.12–1.95, *p* = 0.006, *I*^2^ = 60%; Figure 4]. Consistent results were retrieved in subgroup analyses according to study design (Figure 4A) or adjustment of LDL-C at baseline (Figure 4B). Moreover, a subgroup analysis did not show that differences in the definitions of SIH could significantly affect the association between the premorbid use of statin and the risk of SIH in AIS after IVT (*p* for subgroup difference = 0.29; Figure 4C). A meta-analysis of 10 studies (9, 11, 13, 16–18, 22–24, 28) showed that the premorbid use of statin was not associated with an improved 3-month mortality (OR: 1.12, 95% CI: 0.94–1.34, *p* = 0.20, *I*^2^ = 20%; Figure 5). Subgroup analyses according to study design showed a similar result (Figure 5). None of the 10 studies included in the meta-analysis of 3-month morality adjusted LDL-C level at baseline.

### Publication Bias

**Figure 6:**
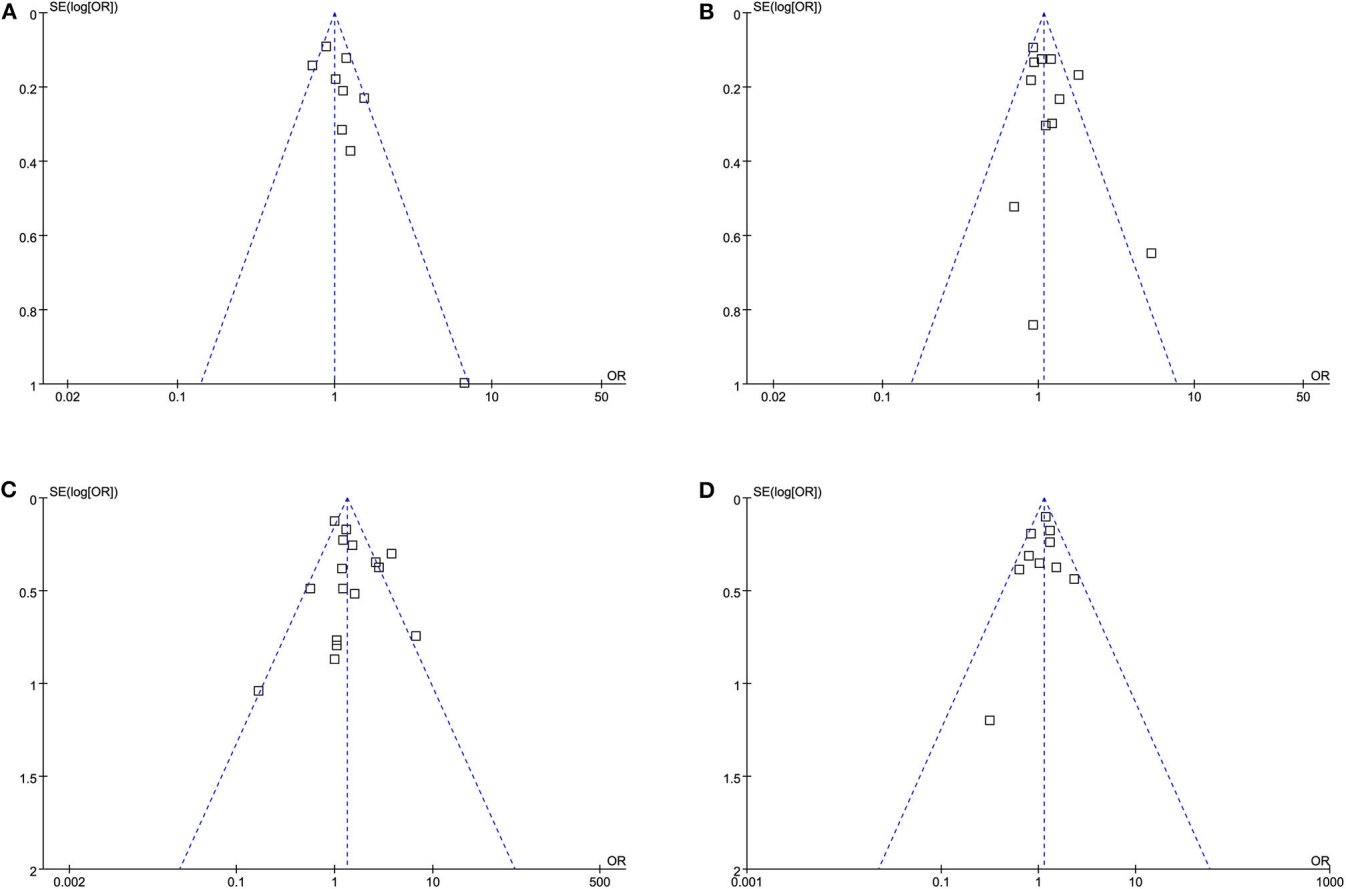
Funnel plots for the meta-analyses: **(A)** 3-month favorable functional outcome, **(B)** 3-month functional independence, **(C)** incidence of symptomatic intracranial hemorrhage, and **(D)** 3-month all-cause mortality.

The funnel plots for the associations between the premorbid use of statin and 3-month favorable functional outcome, 3-month functional independence, incidence of SIH, and 3-month mortality are shown in Figures 6A–D. The plots were symmetrical on visual inspection, suggesting low risks of publication biases. The results of Egger's regression-tests also showed similar results (*p*-values all >0.10).

## Discussion

The main findings of the meta-analysis are that, for AIS patients after IVT, the premorbid use of statin was not associated with an improved functional outcome or survival at 3 months. Moreover, the premorbid use of statin was associated with a higher risk of SIH. These findings are not consistent with the previously observed benefits of the premorbid use of statin on functional outcome and survival as well as the previous finding that the premorbid use of statin did not increase the risk of SIH (3, 6).

To the best of our knowledge, meta-analytic studies focusing on the role of the premorbid use of statin in AIS patients after IVT are rare. Although some previous meta-analyses have evaluated the association between the premorbid use of statin and outcomes after AIS, the potential influences of different canalization treatments, such as thrombolysis and thrombectomy, on the association were generally not considered (3, 5, 6, 34). Our meta-analysis focused on AIS patients after IVT, a group of high-risk patients for SIH, showed that the premorbid use of statin was not associated with an improved functional outcome or survival at 3 months but was related to an increased risk of SIH. Subsequent subgroup analyses showed that the results were consistent in studies of prospective or retrospective design, with or without adjustment of baseline LDL-C and definitions of SIH, which further convinced the robustness of the findings. Although these results were based on observational studies, only those with multivariate analyses were included, aiming to minimize the influence of potential confounding factors on the results. These findings suggest that the premorbid use of statin was not associated with an improved functional outcome or survival but associated with a higher risk of SIH in AIS after IVT.

Previous pre-clinical studies generally supported that the premorbid use of statin could attenuate neurological injury after acute cerebral ischemia *via* mechanisms such as anti-inflammatory and anti-oxidative effects, etc. (4). Clinical studies also showed that statin treatment in AIS may reduce systemic inflammation (35), improve collateral circulation, and reduce infarct volume (36), leading to a lower incidence and burden of microembolic signals during transcranial Doppler monitoring (37). However, clinical trials evaluating the efficacy and the safety of statins in the acute phase of AIS showed inconsistent results. An earlier study in 89 AIS patients showed that, compared to patients randomized to immediate atorvastatin at 20 mg/day, those randomized to statin withdrawal for the first 3 days after admission was associated with an increased risk of death or dependency at 90 days (38). The other pilot study, however, failed to show any short-term benefit of atorvastatin during the acute phase of AIS, while a possible favorable functional effect at 3 months in the least severe strokes was suggested (39). Moreover, this was not supported by another RCT, which showed that treatment with atorvastatin and irbesartan, initiated on day 3 after AIS, did not appear to substantially modify infarct growth (40). Similarly, a recent RCT also failed to show any superiority of early statin therapy within 24 h of admission compared with delayed statin therapy at 7 days after admission to alleviate the degree of disability at 90 days after AIS onset (41). Collectively, evidence based on previous RCTs could not show a conclusive effect of the premorbid use of statin on functional or clinical outcomes in AIS patients. Moreover, besides AIS patients after IVT, these RCTs also included AIS patients treated with thrombectomy. It remains unknown whether the role of the premorbid use of statin differs between patients receiving thrombolysis and thrombectomy. From this perspective, a current meta-analysis focusing on AIS patients after IVT showed that the premorbid use of statin was not associated with any benefits in functional outcome or survival in these patients. Although these findings were based on observational studies and further validation by RCTs is needed, our results suggested that the premorbid use of statin was not likely to be associated with any functional or clinical benefits in these patients.

Moreover, we found that the premorbid use of statin was related to a higher risk of SIH in AIS patients after IVT, which is consistent with previous studies showing that statin use may be related with a higher risk of hemorrhage. A previous study showed that statin users had a higher risk of gastrointestinal hemorrhage than other chronic medication users (42). Moreover, in patients with CVD, a meta-analysis of seven RCTs showed that a higher dose of statins was associated with a risk of intracranial hemorrhage (ICH) (43). Previous studies also showed that a lower serum LDL-C may be associated with a higher risk of ICH (44, 45). However, our subgroup analyses found similar results in studies with and without adjustment of baseline LDL-C level, suggesting that the association between the premorbid use of statin and an increased risk of SIH in AIS after IVT seemed to be independent of LDL-C. More studies are needed to determine whether the interaction between the premorbid use of statin and LDL-C level is involved in the development of SIH in these patients.

Substantial heterogeneity was detected for the meta-analyses of the associations between the premorbid use of statin and 3-month functional outcomes, 3-month mortality, and risk of SIH in AIS after IVT. Although subgroup analyses did not support that difference in study design or adjustment of LDL-C was a potential contributor to heterogeneity, we were unable to determine the source of potential heterogeneity at the current stage. It could be hypothesized that difference in brain imaging characteristics of the AIS patients, etiologies of AIS, and the type, dose, and duration of statins may all contribute to heterogeneity among the included studies. However, we were unable to confirm these hypotheses since these data were rarely reported in the original studies. Besides that, our study also has some limitations. A number of retrospective studies were included, which may confound the findings of the meta-analysis by selection bias and recall bias. However, the subgroup analysis showed consistent results in prospective and retrospective studies. In addition, the confounding factors adjusted in the original studies were varied, which may contribute to the potential heterogeneity. More studies are needed to determine their influences on the outcomes of the meta-analysis.

## Conclusions

In conclusion, this meta-analysis showed that the premorbid use of statin is not associated with an improved functional outcome or survival at 3 months in AIS patients after IVT. Moreover, the premorbid use of statin is associated with an increased risk of SIH in these patients.

## Data Availability Statement

All datasets generated for this study are included in the article/supplementary material.

## Author Contributions

JL and XJ designed the study and drafted the manuscript. JL and QW performed database search, study inclusion, quality evaluation, and data extraction. CY, GL, BZ, and ZJ performed statistical analyses and interpreted the data. All authors critically reviewed the manuscript and approved its submission.

## Conflict of Interest

The authors declare that the research was conducted in the absence of any commercial or financial relationships that could be construed as a potential conflict of interest.
